# A Two-Stage Screening Approach with I-TC and Q-CHAT to Identify Toddlers at Risk for Autism Spectrum Disorder within the Italian Public Health System

**DOI:** 10.3390/brainsci10030184

**Published:** 2020-03-23

**Authors:** Raffaella Devescovi, Lorenzo Monasta, Maura Bin, Giulia Bresciani, Alice Mancini, Marco Carrozzi, Costanza Colombi

**Affiliations:** 1 Division of Child Neurology and Psychiatry, Institute for Maternal and Child Health – IRCCS “Burlo Garofolo”, 34137 Trieste, Italy; maura.bin@burlo.trieste.it (M.B.); giulia.bresciani1993@gmail.com (G.B.); marco.carrozzi@burlo.trieste.it (M.C.); 2 Clinical Epidemiology and Public Health Research Unit, Institute for Maternal and Child Health – IRCCS “Burlo Garofolo”, 34137 Trieste, Italy; lorenzo.monasta@burlo.trieste.it; 3 IRCCS Stella Maris Foundation, Calambrone, 56128 Pisa, Italy; amancini@fsm.unipi.it; 4 Department of Psychiatry, University of Michigan, Ann Arbor, MI 48109, USA; ccolombi@med.umich.edu

**Keywords:** autism spectrum disorder, screening, early detection

## Abstract

Standardized screening programs ensure that children are monitored for early signs of autism spectrum disorder (ASD) in order to promote earlier diagnosis and intervention. The aim of this study is to identify early signs of atypical development consistent with ASD or other developmental disorders in a population of 224 low-risk toddlers through a two-stage screening approach applied at 12 and 18 months of age. We adopted two screening tools combined: 1. the Communication and Symbolic Behavior Scales Developmental Profile (CSBS DP) Infant–Toddler Checklist (I-TC) and 2. The Quantitative Checklist for Autism in Toddlers (Q-CHAT). We assessed their sensitivity and specificity related to the diagnostic outcome at 36 months. The results showed that autistic signs can be detected as early as the first year even through a few questions extrapolated from both screeners and that our model could be used as a screening procedure in the Italian public health system.

## 1. Introduction

Autism spectrum disorder (ASD) is a heterogeneous complex of neurodevelopmental disorders distinguished by impairments in social communication, reciprocal interaction and repetitive pattern of behaviors and interests, as defined by the Diagnostic and Statistical Manual of Mental Disorders, Fifth Edition (DSM-5) [1].

Clear evidence exists that early detection and early intervention can lead to a better prognosis [2,3,4]. According to the latest revision of the American Academy of Pediatrics (AAP) guidelines about promoting optimal development in infants and young children, the early identification of developmental disorders should be conducted through developmental surveillance and periodic screening at each pediatric health visit [5]. The AAP recommends that specific screeners for ASD should be administered to all children at their 18- and 24-month visits because screening tests enhance the accuracy of the developmental surveillance process [6]. On the other hand, many prospective studies investigating siblings of children with ASD, a high-risk population for ASD, showed that early signs of ASD can be identified as early as 12 months of age [7,8]. However, screenings conducted too early may not be able to distinguish ASD from other developmental disorders, which correspond to the majority of false positive cases—or even from typical development [9].

Family and population studies have supplied evidence of a broader autism phenotype (BAP) referring to the presence of subclinical autistic traits in ASD-patient’s relatives and in the general population, such as social-communication deficits and rigidity of personality and behaviors not severe enough to deserve a diagnosis of ASD [10,11,12,13]. It remains unclear whether, in early development, mild social communication deficits and personality rigidity are part of the BAP or they represent early signs of ASD because only a few studies have investigated BAP features in infancy and toddlerhood [14].

Despite the increase of developmental screening tools, it is likely that no single screening test is appropriate for all children at all ages [15]. Repeated and regular screenings may be more effective than a single screening to differentiate properly the early signs of ASD from other developmental conditions [9]. This statement is supported by a recent review containing six studies conducted in Europe on screening procedures and strategies, which suggest that an ASD population screening is more efficacious if it adopts a multi-stage approach and if it combines different screening tools in order to cover a wider range of age and severity of symptoms, thus minimizing the number of false negatives [16].

The aim of our study was to identify early signs of atypical development consistent with autism spectrum disorder (ASD) and broader autism phenotype (BAP) conditions in a population of low-risk toddlers through a two-stage screening approach. We combined two screening instruments for ASD that are not commonly used in the Italian context: 1. the Communication and Symbolic Behavior Scales Developmental Profile (CSBS DP) Infant–Toddler Checklist (I-TC) [17,18,19] and 2. the Quantitative Checklist for Autism in Toddlers (Q-CHAT) [20]. The two instruments were used in a two-stage screening approach at 12 and 18 months of age. Then, we followed the screen positive cases through consecutive evaluations of cognitive, language, motor and social skills until the final diagnostic outcome at 36 months of age. We chose the Q-CHAT questionnaire as a general assessment of autistic traits because it better explores the quantitative differences between ASD and general population; while the I-TC, originally developed for early detection of language delay, was chosen because of its emphasis on pre-linguistic communication and some social components that are key features of early ASD, including gestures and shared attention. Finally, we tried to identify from both screeners the items most sensitive to predict an ASD diagnosis to help clinicians in the referral process for a full diagnostic evaluation.

## 2. Materials and Methods

### 2.1. Study Design

We report data from the administration of two short screening questionnaires: 1. the Quantitative Checklist for Autism in Toddlers (Q-CHAT) and 2. One measure of the Communication and Symbolic Behavior Scales Developmental Profile (CSBS DP), the Infant–Toddler Checklist (I-TC). These screeners were administered to an unselected population of toddlers. The screening protocol required the questionnaires to be administered personally to the parents by a child psychologist at 12 months and repeated at 18 months of age, regardless of the result of the first screening. The questionnaires were administered at 12 months in specialized public health vaccination centers where children received mandatory vaccinations, because, in Italy, vaccinations at 12 months are mandatory, while at 18 months the same psychologist administered the screeners by telephone. All parents agreed to participate in the study on a voluntary basis and provided informed consent. The study was approved by the Technical Scientific Committee of the Institute for Maternal and Child Health-IRCCS “Burlo Garofolo” in Trieste, Italy (Prot. CE/V-151).

Children who screened positive in both questionnaires at 12 months, and only in one of them at 18 months, were evaluated by a child neuropsychiatrist expert in autism who confirmed the ASD risk and recruited to participate in a longitudinal prospective study involving diagnostic evaluation every 6 months (at 12-18-24-30-36 months) from the time of recruitment until 36 months of age. The diagnostic assessment was based on the clinical judgement and standardized tests’ results for cognitive, language, motor and social domains. In case of diagnostic concerns, children were referred for early intervention. Families received diagnostic feedback at each follow-up visit. Moreover, the child’s pediatrician received a letter describing the study prior to the beginning of the study, as well as screening and diagnostic evaluation reports. Data regarding the follow-up evaluations and, consequently, the description of the developmental trajectories will be described in a forthcoming publication, given that the focus of the current publication is on early detection of ASD.

**Figure 1:**
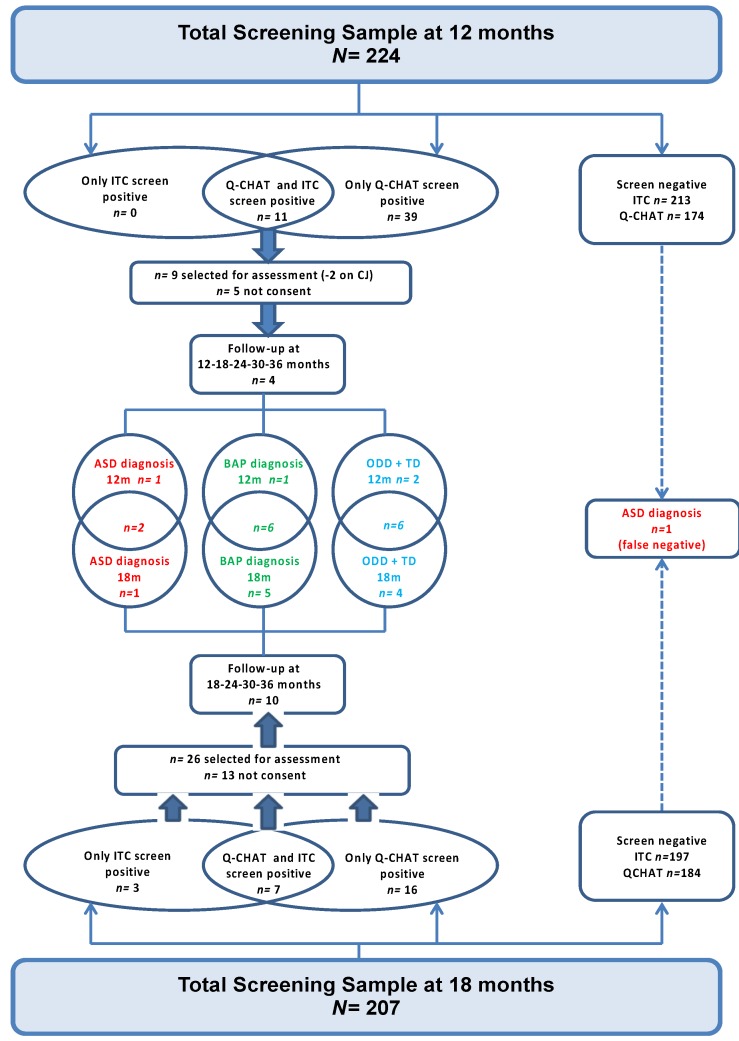
Flowchart of the project design. Project design. Two-stage screening approach at 12 and 18 months applied to the same sample. The intersections in the middle represent the children classified as autism spectrum disorder (ASD) (*n* = 2), broader autism phenotype (BAP) (*n* = 6), Other non-spectrum developmental disorders (ODD) + typical development (TD) (*n* = 5) at the final outcome of 36 months. On the right the only false-negative case diagnosed as ASD at 36 months. I-TC: Infant–Toddler Checklist; Q-CHAT: Quantitative Checklist for Autism in Toddlers.

The flowchart in Figure 1 describes the whole design of the study.

Neurodevelopmental disorders of known genetic etiology and significant vision, hearing, motor or physical problems have been identified as exclusion criteria. Two children were excluded from the study at the 12 months’ data point because they were affected with a genetic disorder characterized by global developmental delays and dysmorphic features. For the diagnostic follow-up evaluations, 9 children at 12 months and 26 children at 18 months were recruited respectively. Among those who respected the recruitment criteria, only 4 out of 9 children at 12 months, and 13 out of 26 children at 18 months were included in the study. Therefore, approximately half of the parents did not consent to the diagnostic assessment; additionally, 3 out of 13 children recruited at 18 months left the study after the 24 months follow-up visit because the parents did not recognize any risk for their child’s development. At the last follow-up visit at 36 months, there were only 14 children who fully participated until the end of the study and received a final diagnosis. ASD diagnosis was confirmed based on the Diagnostic and Statistical Manual of Mental Disorders, 5th Edition (*DSM-5*) criteria [1] and the ADOS-2 [21], administered by experienced clinicians trained in research reliability.

### 2.2. Participants

At 12 months, 224 toddlers were enrolled in the study. Of these, 207 toddlers repeated the screening at 18 months. The outcome at 36 months is known for all the children, even those with negative screenings, because in case of any developmental problems, they would be sent for diagnostic evaluation by their pediatrician at the only diagnostic center in the Trieste area, located at the Division of Child Neurology and Psychiatry of the Institute for Maternal and Child Health— IRCCS “Burlo Garofolo” in Trieste, Italy—a Regional public Institute for Health care and scientific research.

### 2.3. Measures

As screening tools, we used the Infant–Toddler Checklist (I-TC) and the Quantitative Checklist for Autism in Toddlers (Q-CHAT) to identify children at risk for autism spectrum disorder in a low-risk population. We expected to identify children with autistic symptoms or traits consistent with ASD diagnosis or with a BAP condition, versus children with Other non-spectrum Developmental Disorders (ODD) and children with typical development (TD). Children classified as BAP displayed autistic traits below the ASD threshold. The I-TC is a part of the Communication and Symbolic Behavior Scales Developmental Profile (CSBS DP) and is a broadband screener for communication delays of children between 12 and 24 months of age.

The I-TC is a screening questionnaire that investigates children’s social communication through 24 questions clustered in: emotion and eye gaze, communication, gestures, sounds, words, understanding, object use. It can be downloaded from www.brookespublishing.com/resource-center/screening-and-assessment/csbs/csbs-dp/csbs-dp-itc [22]. With a cut off of the 10th percentile relative to population norms, a positive screen indicates risk for communication delay, but it does not discriminate between ASD and other developmental disorders.

The Q-CHAT is a 25-item questionnaire for caregivers testing children’s autistic behaviors and traits in toddlers aged 18 to 24 months. Each Q-CHAT item is scored on a 5-point scale to assess frequency, typicality and severity of autistic behavior, through a dimensional-quantitative approach.

We chose the cut-off the score as 38 for both 12 and 18 months because, in Allison et al. [20], 80% of children with ASD had a cut-point of at least 38% versus 8% of children with typical development. Both screening tools have been translated into Italian with the back-translation mode.

The diagnostic assessment included a clinical observation conducted by the child neuropsychiatrist as well as the administration of the following diagnostic tools:

The *Autism Diagnostic Observation Schedule, Second Edition* (ADOS-2) is a semi-structured schedule that investigates different areas of ASD, including social communication, play and repetitive behaviors. In addition to the clinical judgment, ADOS-2 distinguishes between ASD and other delays or typical development. This instrument was used as a part of the diagnostic evaluation.

The *Bayley Scales of Infant and Toddler Development, Third Edition* [23] evaluates cognitive, language and motor skills in children between 0 and 42 months. This instrument was used as a part of the diagnostic evaluation. 

### 2.4. Statistical Analysis

We carried out descriptive analyses in order to present the characteristics of the population considered. We subsequently carried out bivariate logistic regressions, considering positivity to ASD or BAP at 36 months of age as outcome and single Q-CHAT items and I-TC clusters as potential predictors, collected at 12 and 18 months of age. We also considered the summary scores resulting from the Q-CHAT and I-TC, both at 12 and 18 months of age, as potential predictors. Finally, we conducted two separate multivariate logistic regressions with Q-CHAT items and I-TC clusters, respectively, that resulted in significant association with the outcome at bivariate logistic regression. We, then, adopted a stepdown procedure in order to obtain two potentially predictive models, one with Q-CHAT items and the other with I-TC clusters. For each of these final models, we also generated Receiver Operating Characteristic (ROC) curves, calculated the respective Areas Under the Curves (AUC) and selected sensitivity and specificity cut-offs. All statistical analyses were performed using Stata/IC14.2 (Stata/IC 14.2 (StataCorp LLC, College Station, TX, USA).

## 3. Results

At the final diagnostic assessment of 36 months, we have identified three children with ASD and six children with BAP, three children with ODD (i.e., language delay) and three others with TD. Two out of the three children diagnosed with ASD were identified through the screening. The third child who had scored 37 at Q-CHAT at 18 months was a false negative at screening and was identified by his pediatrician and referred later to the autism evaluation center for diagnostic evaluation. The sample consisted of 224 children (female = 50%, *n* = 113; male = 50%, *n* = 111). 

As shown in Table 1, the majority of parents held a high school diploma or higher educational qualification (mothers: 85%, *n* = 191; fathers: 85%, *n* = 191), with 53% of the mothers (*n* = 119) and 39% of the fathers (*n* = 88) holding at least a bachelor’s degree. Seventy percent of the mothers (*n* = 157) and 96% of the fathers (*n* = 215) were employed at the time of the study.

We analyzed the properties of the two screeners in terms of sensitivity, specificity, positive predictive value (PPV) and negative predictive value (NPV). We have dichotomized the sample into two groups: the ones with TD and ODD (called non ASD) and the ones with ASD diagnosis or BAP conditions (called ASD), as shown in Table 2.

At 12 months, we found that the specificity was high for both screeners, better for I-TC (96%) than Q-CHAT (80%), while the sensitivity was low for both, better for Q-CHAT (67%) compared to I-TC (22%). The value of PPV was slightly higher in I-TC (18%) compared to Q-CHAT (12%), whilst the percentage of NPV remained high for both I-TC (97%) and Q-CHAT (98%). 

At 18 months, we found that the specificity remained high in both the screeners, equally in Q-CHAT (92%) and I-TC (98%), while the sensitivity increased moderately in both with a greater extent in Q-CHAT (78%) than the I-TC (67%). The PPV increased in I-TC (60%) and Q-CHAT (30%) and NPV remained high (I-TC: 98%; Q-CHAT 99%).

At this point, we tried to identify both for Q-CHAT and I-TC items and clusters that are more often associated with ASD diagnosis or BAP conditions at the final 36 months’ outcome. We found that at 12 months, through a bivariate logistic regression analysis, 5 items of the Q-CHAT were significantly associated with positivity to ASD or BAP (i.e., 5, 6, 10, 19 and 20; *p* < 0.05). These items were considered in a multivariate logistic regression analysis; through a stepdown procedure, by eliminating non-significant items with the higher p-value one at the time, we obtained a model with only 3 statistically significant items: item 6 (“*Does your child point to share interest with you (e.g., pointing at an interesting sight)*?”), item 19 (“*Does your child use simple gestures (e.g., wave goodbye)?*”) and item 20 (“*Does your child make unusual finger movements near his/her eyes?*”) (Table 3).

**Figure 2:**
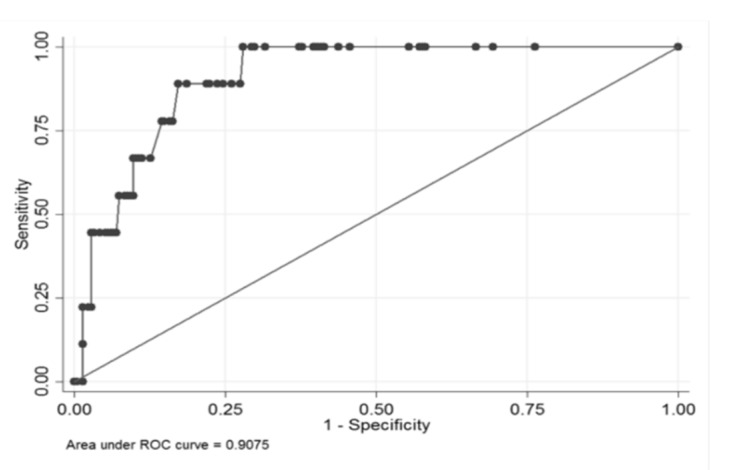
Area under the receiver operating characteristic (ROC) curve (AUC) of 90.7%.

This model had an area under the receiver operating characteristic (ROC) curve (AUC) of 90.7% and a cut-off could be chosen with 100% sensitivity and 72% specificity (Figure 2).

At 18 months, the bivariate logistic regression analysis allowed us to identify 17 Q-CHAT items that were significantly associated to positivity to ASD or BAP (1, 2, 3, 4, 5, 6, 7, 8, 9, 10, 14, 15, 16, 17, 19, 20, 23 and 25; *p* < 0.05). Again, starting from these items, we carried out a multivariate logistic regression adopting a stepdown procedure and obtained a model with 4 significantly associated items: item 10 (“*Does your child follow where you’re looking?*”), item 14 (“*How easy is it for your child to adapt when his/her routine changes or when things are out of their usual?*”), item 19 (“*Does your child use simple gestures (e.g., wave goodbye)?*”), item 20 (“*Does your child make unusual finger movements near his/her eyes?*”) (Table 4).

**Figure 3:**
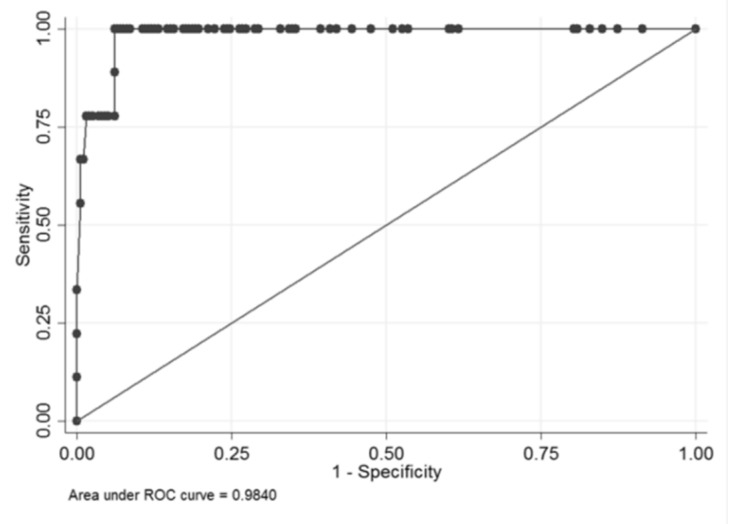
Area under the receiver operating characteristic (ROC) curve (AUC) of 98.4%.

The final model had an AUC of 98.4%; we could keep an 100% sensitivity with a 93.9% specificity (Figure 3).

Regarding the I-TC, the bivariate logistic regression analysis identified only one cluster at 12 months that was significantly associated with positivity to ASD or BAP: cluster 2 (Communication) (Odd ratio= 0.53; C.I. 95% = 0.009; *p*-value = 0.33–0.85). At 18 months, all seven I-TC clusters were significantly associated (*p* < 0.05). In the multivariate logistic regression model, after the application of the stepdown procedure, two clusters resulted in a significant association with the outcome: 1 (emotion and Eye Gaze) and 5 (Words) (Table 5).

**Figure 4:**
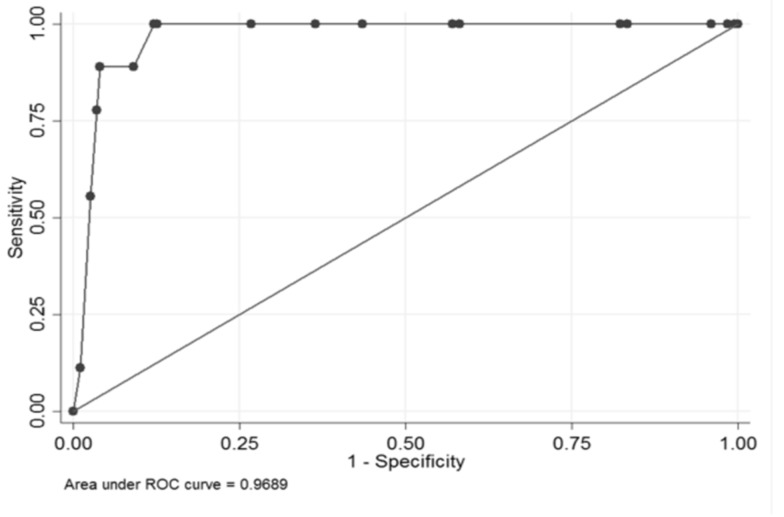
Area under the receiver operating characteristic (ROC) curve (AUC) of 96.9%.

This model had an AUC of 96.9% and maintaining a sensitivity of 100% could reach a specificity of 88% (Figure 4).

**Figure 5:**
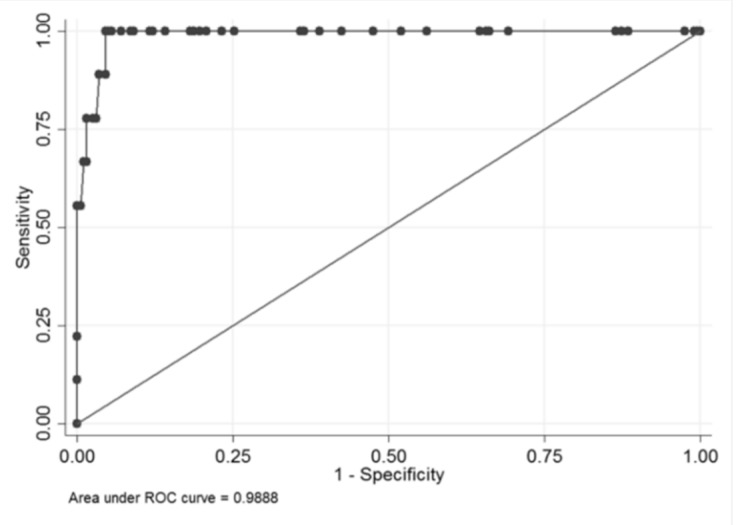
Area under the receiver operating characteristic (ROC) curve (AUC) of 98.9%.

Finally, in a multivariate logistic regression, we combined the statistically significant clusters and items at bivariate logistic regression from I-TC and Q-CHAT at 18 months (Q-CHAT items: 1, 2, 4 to 10, 14 to 17, 19, 20, 23 and 25; I-TC clusters: 1 to 7) and run a stepdown procedure. The model we obtained was based on three “predictors”: I-TC clusters 1 and 5 and Q-CHAT item 20. This model had an AUC of 98.9% and obtained 100% sensitivity with and 95% specificity (Figure 5).

We did the same analysis combining the statistically significant clusters and items at bivariate logistic regression from I-TC and Q-CHAT at 12 months, to run a stepdown procedure (Table 6).

However, the significant clusters from I-TC were the first to be excluded, thus the results were solely based on Q-CHAT items as in the previously exposed model, shown in Table 4.

## 4. Discussion

We have described a screening protocol applied to a population of low-risk toddlers recruited at the clinics where mandatory vaccinations are carried out. Two different screeners were both administered at 12 and 18 months of age to identify the signs of risk for autism. Of all the children, we could know the outcome at 36 months because those who tested positive at 12 and at 18 months were longitudinally evaluated while any false negatives would have been referred by their pediatrician to the only available diagnostic center in the area. Therefore, we can affirm with reasonable certainty that, due to the screening carried out at two distinct stages, we were able to identify one case of ASD at 12 months and another one at 18 months. The third case of ASD was, unfortunately, the false negative who scored below 38 in the Q-CHAT and would likely be avoidable if we had adopted a risk “range” rather than using a pre-established cut-point. However, we made this choice based on data published by Allison et al. [20] in order to avoid recruitment of too many false positive cases.

We found that, at the age of 12 months, neither the Q-CHAT nor the I-TC has good overall sensitivity while, at 18 months, only the Q-CHAT has good sensitivity. Surely, more interesting was the result of the analysis that allowed us to identify some Q-CHAT items and some I-TC clusters, statistically more significant than the other items at 12 and 18 months, respectively. Of these two screening tools, we analyzed the properties and selected some items and clusters of items that are more sensitive to diagnostic identification. Such clusters may represent a brief measure to help determine whether a full diagnostic evaluation is needed. 

In particular, the Q-CHAT has three items at 12 months and four items at 18 months with a very high sensitivity, which correspond to those questions that investigate the shared attention (items 1 and 6), the presence of simple communicative gestures (item 19) and the presence of stereotypical movements with fingers close to the eyes (item 20). Specifically, these last two items, items 19 and 20, remain significant at both 12 and 18 months as they maintain very high sensitivity and specificity at both ages. This finding would support previous research describing the presence of repetitive behaviors among children who go on to develop ASD as early as 12 months of age [24].

Regarding the I-TC instead, at 12 months, the only cluster significantly related to the outcome is communication (cluster 2) while, at 18 months, the clusters investigating areas, such as social engagement and shared attention (cluster 1) and verbal communication (cluster 5) appear. Moreover, at 18 months, these last two clusters of the I-TC, combined with item 20 of the Q-CHAT, constitute a model of three predictors with very high sensitivity and specificity. This confirms that the I-TC is a broadband screener which covers multiple developmental areas while the Q-CHAT seems more specific for autism and better discriminates among autism children, typical development and also from other developmental conditions, as suggested by Ruta et al. [25].

Also in our study it appears evident that it is more difficult to identify at 12 months any screening tools—or single items—that maintain a stable predictive value. This is the reason why we established as a recruitment criterion at 12 months that toddlers were positive for both screeners and that they were visited by a neuropsychiatrist expert in autism, in order to obtain a Clinical Best Estimate (CBE); while at 18 months being positive for only one of the two screeners, confirmed by clinical judgment, was enough. In this way, we were able to identify already at 12 months one out of three of the children who were diagnosed with ASD at the following diagnostic assessments and thus sent him for early intervention. The recruitment strategy we adopted in our study could be recommended in order to limit the rate of false cases, which is certainly higher at 12 months than at 18 months. Furthermore, our results suggest that it would be possible to administer at 12 months only the three most sensitive Q-CHAT items and at 18 months the short version of three predictors to identify a risk for ASD, being aware that in very young children (12–14 months) it is correct to assume a risk; it is not yet possible to make a diagnosis. However, these results seem to be promising and worthy of future confirmation in larger studies.

In our model, we believe that the screening combined with a mandatory procedure, due to vaccination policies in Italy, can optimize the spread of screening to a wider low-risk population. In addition, as an unexpected consequence, a large majority of parents declared that they had been given an educational opportunity and felt that they had gained a greater awareness in monitoring their child’s development. Perhaps, this active participation by parents could have been positively influenced by the high level of parents’ education, especially of mothers (as can be seen from the socio-demographic data table). Moreover, the repetition of screening helps to identify a wider population at developmental risk composed of children with late onset of symptoms and false positive cases with other neurodevelopmental disorders. Screening conducted too early may not be able to distinguish ASD from other developmental delays or even typical developmental delays as it may not detect cases of plateau or regression, which are about 30% of individuals with ASD [26,27]. Only a longitudinal diagnostic assessment can confirm the ASD diagnosis and provide major details about the different developmental trajectories [28,29]. Additionally, in case of false positives, which often result in other non-spectrum disorders, early recognition can mean a better prognosis and earlier access to treatment. Among these cases, we also include BAP, which is not a diagnostic entity due to much milder difficulties than ASD. However, BAP in early childhood has been described as social and communication difficulties and rigidity of behaviors; little or nothing is known about its long-term evolution. We can hypothesize that subtle ASD signs at early ages could become more evident at school age under an increasing social demand [30]. Therefore, it is crucial to know more about the long-term consequences of certain early developmental patterns and to provide guidance to parents.

Our study presents some limitations including a small sample size and a limited geographical area. Therefore, our study should be replicated with a larger sample size in a larger geographical area. Despite the limitations, if our results are confirmed with a bigger sample, our model could be used as a screening procedure in the Italian public health system.

In conclusion, the results of our study show that atypical aspects of development can be identified as early as the first year of life and that two different screening tools, such as the I-TC and the Q-CHAT, combined together and administered in a two-stage approach can help to identify children at risk for ASD symptoms or autistic traits, perhaps even using reduced versions consisting of a few questions extrapolated from both screeners.

## Figures and Tables

**Table 1:** Socio-demographic characteristics of the sample (*n* = 224).

Variables.	Modalities	Mean (SD) or Number (%)
Sex, *n* (%)	Males	111 (50)
	Females	113 (50)
Prematurity *n* (%)		15 (7)
Twins *n* (%) twin birth		4 (2)
Kindergarten attendance, *n* (%)		64 (29)
Maternal age at delivery, years, mean (SD)		32.7
Paternal age at delivery years, mean (SD)		36.1
Maternal educational level, *n* (%)	Elementary school	1 (0.4)
	Middle school	32 (14)
	High school	72 (32)
	University degree	119 (53)
Paternal educational level, *n* (%)	Elementary school	1 (0.4)
	Middle school	32 (14)
	High school	103 (46)
	University degree	88 (39)
Maternal occupational status, *n* (%)	Employed	157 (70)
	Housewife	63 (28)
	Other/missing	4 (2)
Paternal occupational status, *n* (%)	Employed	215 (96)
	Unemployed	2 (1)
	Other/missing	7 (3)

SD: standard deviation.

**Table 2:** Contingency tables of positivity to ASD and BAP at 36 months and positivity to I-TC and Q-CHAT at 12 and 18 months.

	12 Months (*n* = 224)				
	I-TC		Q-CHAT		
	Negative	Positive	Negative	Positive	
Non ASD	206 (96%)	9 (4%)	171 (80%)	44 (20%)	215 (100%)
ASD	7 (78%)	2 (22%)	3 (33%)	6 (67%)	9 (100%)
	**18 Months (*n* = 207)**				
Non ASD	194 (98%)	4 (2%)	182 (92%)	16 (8%)	198 (100%)
ASD	3 (33%)	6 (67%)	2 (22%)	7 (78%)	9 (100%)

At 12 months. I-TC: Sensitivity 22%; Specificity 96%; Positive predictive value 18%; Negative predictive value 97%. Q-CHAT: Sensitivity 67%; Specificity 80%; Positive predictive value 12%; Negative predictive value 98%. At 18 months. I-TC: Sensitivity 67%; Specificity 98%; Positive predictive value 60%; Negative predictive value 98%. Q-CHAT: Sensitivity 78%; Specificity 92%; Positive predictive value 30%; Negative predictive value 99%.

**Table 3:** Results of the multivariate logistic regression analysis stepdown procedure on the association between diagnosis of ASD or BAP at 36 months and Q-CHAT items significantly associated at the bivariate logistic regression at 12 months of age (Items 5, 6, 10, 19 and 20).

Q-CHAT Items	Regression Coefficients	Odds Ratios	95% CI	*p*-Value
6	0.6480598	1.91	1.11–3.30	0.020
19	0.6180474	1.86	1.09–3.15	0.022
20	0.6285134	1.87	1.05–3.35	0.034
constant	−5.987096			

C.I: Confidence Interval.

**Table 4:** Results of the multivariate logistic regression analysis stepdown procedure on the association between diagnosis of ASD or BAP at 36 months and Q-CHAT items significantly associated at the bivariate logistic regression at 18 months of age (Items 1, 2, 4, 5, 6, 7, 8, 9, 10, 14, 15, 16, 17, 19, 20, 23 and 25).

Q_CHAT Items	Regression Coefficients	Odds Ratios	95% CI	*p*-Value
10	1.141131	3.13	1.03–9.52	0.044
14	3.942781	51.56	2.29–1161.08	0.013
19	2.510013	12.31	1.77–85.38	0.011
20	3.062373	21.38	2.40–190.41	0.006
constant	−17.08924			

**Table 5:** Results of the multivariate logistic regression analysis stepdown procedure on the association between diagnosis of ASD or BAP at 36 months and I-TC items significantly associated at the bivariate logistic regression at 18 months of age (Items 1 to 7).

I-TC Items	Regression Coefficients	Odds Ratios	95% CI	*p*-Value
1	−2.103662	0.12	0.03–0.43	0.001
5	−1.62435	0.20	0.06–0.64	0.007
constant	13.96184			

**Table 6:** Results of the multivariate logistic regression analysis stepdown procedure on the association between diagnosis of ASD or BAP at 36 months and I-TC and Q-CHAT items significantly associated in the final models at 18 months of age (I-TC Items 1 and 5 and Q-CHAT Items 10, 14, 19 and 20).

Items	Regression Coefficients	Odds Ratios	95% CI	*p*-Value
I-TC item 1	−2.969194	0.05	0.01–0.41	0.005
I-TC item 5	−2.012009	0.13	0.03–0.71	0.018
Q-CHAT item 20	2.089171	8.08	1.86–35.1	0.005
constant	17.09517			
